# Intra‐amniotic umbilical vein varix: A case report and review of the literature

**DOI:** 10.1002/ijgo.14525

**Published:** 2022-11-23

**Authors:** Basel H. Nasser, Daher Hamid, Yacoub Zakharian, Jimmy E. Jadaon

**Affiliations:** ^1^ Nazareth Hospital, Department of Obstetrics and Gynecology, Affiliated with The Azrieli Faculty of Medicine Bar‐Ilan University Ramat Gan Israel; ^2^ The Azrieli Faculty of Medicine Bar‐Ilan University Ramat Gan Israel

**Keywords:** antepartum assessment, diagnostic ultrasound, obstetric complication, ultrasound Doppler

## Abstract

An umbilical vein varix is a rare fetal condition and is defined as a focal dilatation of the umbilical vein.

## 1 INTRODUCTION

An umbilical vein varix is defined as focal dilatation of the umbilical vein. Most cases of fetal umbilical vein varix are reported to be intra‐abdominal. Extra‐abdominal or intra‐amniotic umbilical vein varix is rare, with only a few case reports published in the literature.

Adverse fetal complications have been reported, including compression and kinking of the umbilical cord, aneurysm rupture, and thrombosis inside the aneurysm.

Owing to the rarity of this condition, there are no specific guidelines for its management during pregnancy, especially follow‐up intervals, or at the time of delivery.

## 2 CASE REPORT

We present the case of a 39‐year‐old woman with her sixth pregnancy who was admitted to our department at 35^+5^ weeks of pregnancy. Her obstetrical history included four term vaginal deliveries and one spontaneous abortion at the fifth week of pregnancy. Her medical and family histories were normal.

This study was approved by the ethics committee of Nazareth Hospital EMMS and the patient gave her consent for us to publish our case findings.

Close prenatal surveillance was performed because of morbid obesity and gestational diabetes mellitus. The second‐trimester triple marker test revealed a 1/271 risk for trisomy 21; amniocentesis for karyotyping was recommended, but the woman refused to consent. An anomaly scan at 22 weeks of pregnancy was normal.

**FIGURE 1:**
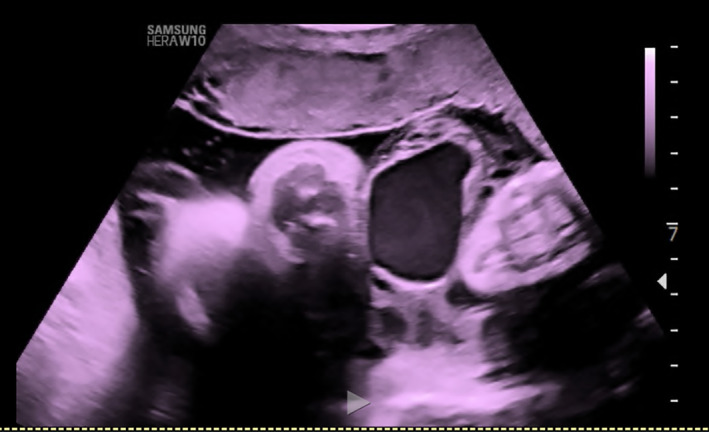
Ultrasound image of 36 weeks gestation shows abnormal dilation of the umbilical vein (intra‐amniotic vein varix).

**FIGURE 2:**
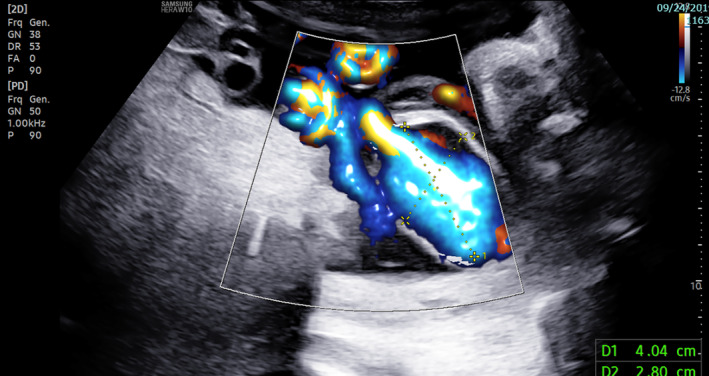
Ultrasonography image shows enlargement of the intra‐amniotic vein varix to 40.4 × 28 mm in the transverse section with color doppler.

**FIGURE 3:**
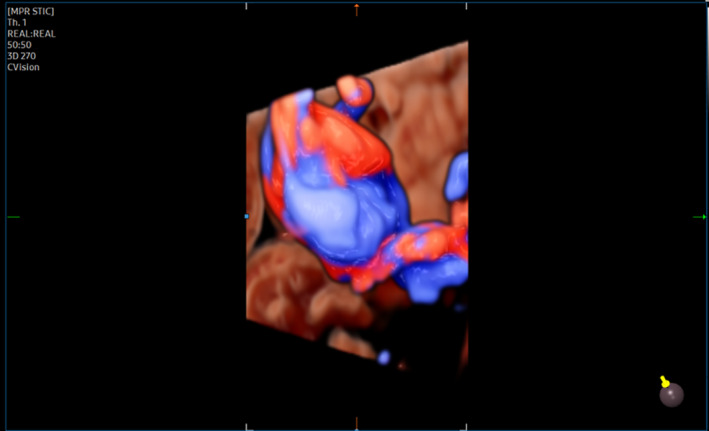
Three‐dimensional ultrasound imaging with color doppler shows turbulence flow in the intravascular area of the intra‐amniotic vein varix.

**FIGURE 4:**
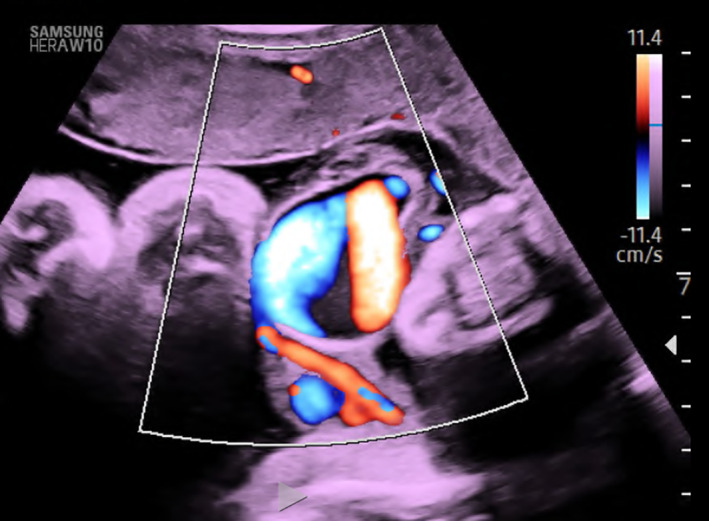
Ultrasound imaging with color doppler shows turbulence flow in the intravascular area of the intra‐amniotic vein varix.

Routine ultrasound performed on admission (35^+5^ weeks) showed the fetus with a vertex presentation. The estimated fetal weight was 2830 g (62nd centile), with a normal biophysical profile. Focal intra‐amniotic dilatation of the umbilical vein was observed with a dimension of 28 × 33 mm in the transverse section but without turbulent flow within the varix. The umbilical artery, middle cerebral artery, and ductus venosus Doppler findings were normal. At 36^+2^ weeks a detailed ultrasound was performed, including fetal echocardiography, which was normal. However, the varix was enlarged to 40.4 × 28 mm in the transverse section (Figures 1 and 2), and turbulence in the varix was observed (Figures 3 and 4).

As a result of the enlargement of the varix and the appearance of turbulent flow, we decided to deliver the premature baby after administration of betamethasone to the patient. After explaining the rarity of this finding and the lack of recommendations in the literature about the mode of delivery, the patient decided to deliver via cesarean section.

**FIGURE 5:**
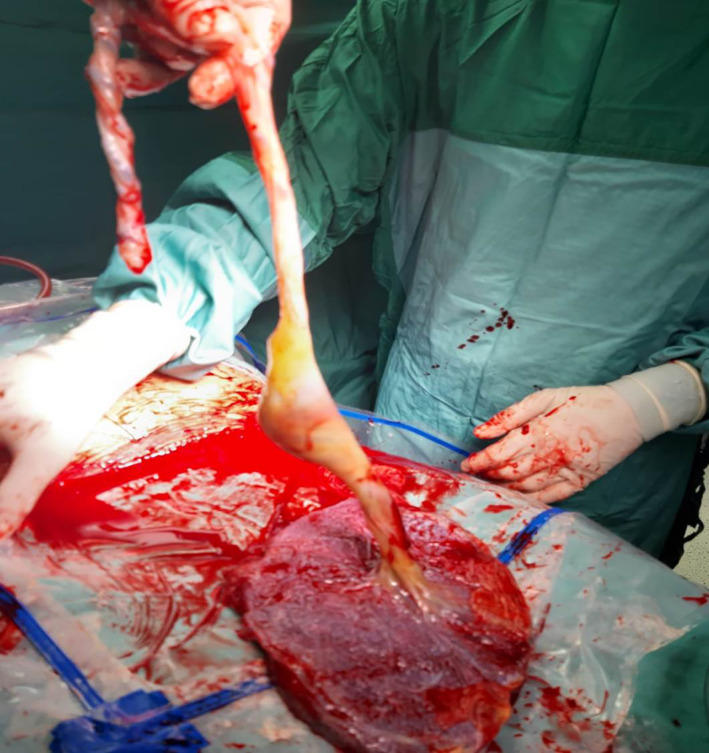
A photograph depicting a placenta with umbilical cord focal dilation.

**FIGURE 6:**
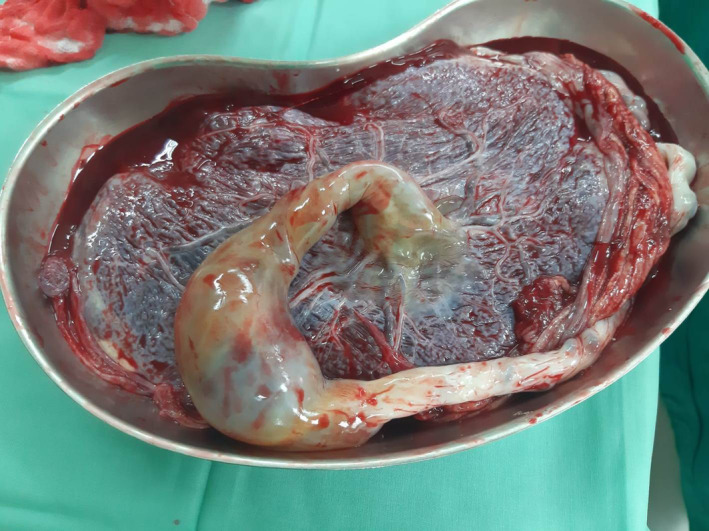
Umbilical cord with focal vein dilation of 60 mm in diameter.

At 36^+4^ weeks, we performed a cesarean section and bilateral tubal ligation as requested. A healthy male baby was born, and the umbilical arterial pH was 7.36. The umbilical cord and placenta (Figures 5 and 6) were sent for histologic examination.

Histopathologic examination showed a 43‐cm‐long, trivascular umbilical cord with focal vein dilatation of 60 mm diameter measured in the transverse section with central insertion to the placenta. The cross‐section of the placenta revealed intra‐placental infarcts with diffuse excess syncytial knots. The placenta had a diameter of 18 × 15 cm, with a maximum thickness of 4 cm and a weight of 662 g (Table 1).

**TABLE 1:** Summary of published case reports of intra‐amniotic vein varix

First author	GW at diagnosis	Delivery GW	Anomalies	Aneuploidy	UC length	Fetal outcome	Delivery
Ghosh (1984)^8^	37	37	NA	No	100	Stillbirth	Vaginal
Vesce (1987)^9^	34	36	No	No	40	Birth	CS
Schrocksnadel (1991)^10^	After delivery	term	NO	No	70	Stillbirth	NA
White (1994)^11^	32	35	SGA	NA	NA	Birth	NA
Shipp (1995)^12^	24.5	34	VSD, dilated SVC	NA	NA	Birth	NA
Vandevijver (2000)^13^	After delivery	41	No	NA	50	IUFD	Vaginal
Berg (2001)^14^	34	34	IUGR, Av fistula	Trisomy 18	NA	Stillbirth	Vaginal
Cruise (2002)^15^	24	32	Klippel‐Trenaunay‐Weber syndrome	NA	NA	IUFD	NA
Zachariah (2004)^17^	After delivery	41	No	No	NA	Birth	Vaginal
Trobs (2012)^16^	27	35	DFG, SGA, and dilatation of left iliac vein	No	NA	Birth	CS
Akar (2012)^18^	32	TERM	No	NA	NA	Birth	CS
Kanenishi (2013)^7^	35	35	No	No	NA	Birth	CS
Jae hoon lee (2014)^2^	34	35	No	NA	35	Birth	CS
Deront‐bourdin (2014)^6^	31	34	No	NA	NA	Birth	CS
Schwaerzker (2016)^19^	20	33	SGA, omphalocele, anemia	no	NA	Birth	CS
Soriano (2015)^3^	36	36	NA	NA	NA	Birth	CS
Alessandro feola (2017)^1^	39	39	no	NA	55	IUFD	CS
Luis Humberto (2017)^4^	38	38	No	No	NA	IUFD	Vaginal
Yuuki Matsumoto (2019)^5^	23	32	No	No	NA	Birth	CS
The present study	35.5	36.4	No	No	43	Birth	CS

Abbreviations: CS, Cesarean section; GW, gestational week; IUFD, intrauterine fetal death; IUGR, intrauterine growth restriction; NA, not announced; SGA, small for gestational age; SVC, superior vena cava; VSD, ventricular septal defect.

The baby was discharged without complications 6 days after birth and was normal at the 6‐month follow up.

## 3 DISCUSSION

We present a rare case of a fetus with an intra‐amniotic umbilical vein varix that was diagnosed and delivered in a late preterm infant with good perinatal outcomes.

Varix of the umbilical cord is a dilatation that can exist in any area across the umbilical cord or the hepatic part of the umbilical vein. It is a rare phenomenon with no known reasons. Doppler examination demonstrated turbulent flow within the aneurysm. Complications of the umbilical cord vein varix can cause fetal morbidity when thrombosis causes blockage of blood flow in the fetus and, consequently, fetal death.

To our knowledge, about 274 cases of fetal intra‐abdominal umbilical vein varix and 19 cases of intra‐amniotic umbilical vein aneurysms have been reported in the English‐language literature. Five of the 19 reported cases underwent vaginal delivery, including seven cases of intrauterine fetal death resulting from occlusion of the umbilical vein due to thrombosis and other anomalies.

The mean gestational age at the time of diagnosis of intra‐amniotic umbilical vein varix and time of delivery were 31^+5^ and 35 weeks of pregnancy, respectively.

Umbilical vein varix is also associated with congenital anomalies, cardiovascular anomalies, imperforate anus, congenital soft‐tissue disease (Klippel‐Trenaunay‐Weber syndrome), and trisomy 18.

In this case report, we describe an aneurysm of the umbilical cord vein that dilated rapidly during 4 days of follow up, with the appearance of turbulent flow. There are no guidelines in the literature regarding the follow up of these cases due to increased fetal morbidity, with few cases of fetal mortality described in previous studies. Therefore, we promoted delivery to avoid fetal death.

We suggest a close follow up, including fetal monitoring and ultrasound follow up, for signs of increased dilatation and the appearance of turbulent flow in the umbilical vein. The timing of delivery remains a challenging decision in the preterm period; however, we suggest considering delivery in late preterm infants in the presence of any suspicious signs that could cause fetal distress.

## AUTHOR CONTRIBUTIONS

The study was designed and planned by Basel H. Nasser, Jimmy E. Jadaon, Yacoub Zakharian, and Daher Hamid, who also analyzed and interpreted the data, and wrote and revised the manuscript. All the authors read and approved the final manuscript.

## CONFLICTS OF INTEREST

The authors have no conflicts of interest to declare.

## Data Availability

The data that supports the findings of this study are available in the supplementary material of this article.
